# The detail is more pleasant than the whole: Global and local prime affect esthetic appreciation of artworks showing whole-part ambiguity

**DOI:** 10.3758/s13414-020-02093-0

**Published:** 2020-07-19

**Authors:** Maddalena Boccia, Paola Guariglia, Laura Piccardi, Giulia De Martino, Anna Maria Giannini

**Affiliations:** 1 Department of Psychology, “Sapienza” University of Rome, Via dei Marsi, 78, 00185 Rome, Italy; 2 Cognitive and Motor Rehabilitation and Neuroimaging Unit, IRCCS Fondazione Santa Lucia, Rome, Italy; 3 Department of Human Science and Society, University of Enna “Kore”, Enna, Italy

**Keywords:** Perceptual ambiguity, Esthetic attitude, Neuroesthetic, Perceptual preference, Global-local perception

## Abstract

Esthetic experience is the result of the coordination of different cognitive processes. It has been widely reported that top-down processes of orienting of attention interact with bottom-up perceptual facilitation occurring during esthetic experience of artworks. Here we use whole-part ambiguity as a tool to test the effect of global and local prime on esthetic appreciation of complex visual artworks. To this aim 139 healthy young individuals completed an esthetic judgment of Arcimboldo’s ambiguous artworks, which were preceded by a local or global prime. Their perceptual style was also assessed using a Navon task. We found that local prime significantly enhanced esthetic appreciation of ambiguous portraits. Also, we found that prime level interacted with individual’s perceptual style: participants showing local perceptual style liked less ambiguous portraits when they were preceded by global prime. Overall, the present findings shed some light on the processes involved in esthetic experience, pointing towards a pivotal role of re-direction of attention towards perceptual features of the artworks and its interaction with individual factors, such as perceptual style.

## Introduction

Esthetic attitude has been proposed to involve an intentional shift from an automatic visuo-perceptual processing to an “esthetic state of mind,” which is more explicitly directed towards the sensory experience (Cupchik, 1992; Cupchik & Winston, 1996). Cela-Conde and colleagues (Cela-Conde et al., 2013) proposed that the esthetic attitude consists mainly of two distinct cognitive events, which take place at different time spans: an initial general appraisal of the esthetic qualities (i.e., the perception of a visual stimulus as beautiful or not), which the authors call “esthetic appreciation sensu stricto” and a delayed appraisal of detailed aspects of the esthetic experience (i.e., whether it is interesting or original), which the authors call “esthetic appreciation sensu lato”. This idea is consistent with evidence from neuroimaging studies. Indeed, the esthetic appreciation sensu stricto has been found to rely on a network of areas encompassing occipital and frontal regions. Instead, the esthetic appreciation sensu lato mainly involves the activation of the default mode network (Cela-Conde et al., 2013).

Based on visual neuroscience studies, Chatterjee (2004) developed a theoretical model of visual esthetic experience. First, all of the elementary visual features of artworks are processed, just as with all other visual objects. Second, attentional processes redirect information processing to the prominent visual properties, such as color, shape, and composition, by means of the fronto-parietal attentional network. Third, the attentional networks modulate processing within the ventral visual stream, which leads to the attributional networks. Specifically, the content of artworks is processed by the attributional areas of the ventral visual stream. Evidence from neuroimaging studies strongly supports the idea that the esthetic appreciation of different categories of paintings is associated with distinct, specialized visual areas of the brain (Boccia et al., 2016): esthetic experience of portraits is associated with activation in brain areas involved in face perception, namely fusiform face area, amygdala, and inferior occipital gyrus; instead, esthetic experience of landscapes yields to activation in the brain areas involved in perceiving scenes, namely parahippocampal place area, lingual gyrus, and retrosplenial cortex. Fourth, feedback/feedforward processes, linking attentional and attributional circuits, enhance the experience of the visual objects. The interaction between attentional and attributional networks during esthetic appreciation has been demonstrated by a study in which esthetic experience was studied as a function of the interaction between state of mind and perceptual facilitation: Cupchik et al. (2009) found that different neural networks subtend a pragmatic and an esthetic attitude toward the artworks, and that different brain areas underpin the esthetic orientation in relation to different perceptual features. According to this evidence, esthetic experience is the result of the interaction between top-down orienting of attention and bottom-up perceptual facilitation. Finally, in most cases, emotional systems are involved. Thus, esthetic experience is not independent from sensory, perceptual, and cognitive processes. Instead, it is interwoven into these processes, as has also been suggested by neuroimaging studies (Boccia et al., 2016).

**Fig. 1:**
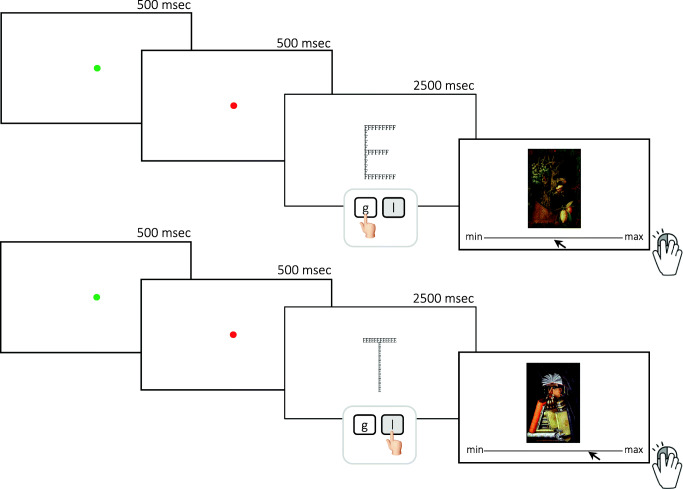
Experimental task: Timeline and examples of stimuli. *Notes* g = global; l = local

Whole-part ambiguity in artworks offers a good tool to investigate the interaction between attributional and attentional processes during esthetic appreciation. Usually, perceptual ambiguity is defined as the stimulus quality of being open to more than one interpretation, which leads to perceptual oscillation (Yevin, 2006) from one percept to another (Andrews, Schluppeck, Homfray, Matthews, & Blakemore, 2002; Kleinschmidt, Büchel, Zeki, & Frackowiak, 1998). Part–whole ambiguity, which is a specific case of perceptual ambiguity, leads to two possible perceptual interpretations, too. It is the main feature of the collection of Arcimboldo’s portraits, which can be interpreted as an array of objects (i.e., fruits, books, flowers) or a face (for examples see Winter and Librarian in Fig. 1). Both behavioral (Boccia et al., 2014) and neuroimaging (Boccia et al., 2015) investigations support the idea that esthetic appreciation of Arcimboldo’s ambiguous artworks is prompted by appreciation of local parts: individuals with local perceptual style, namely those who were faster in detecting the local level of hierarchical stimuli such as Navon letters (Navon, 1977), highly appreciated Arcimboldo’s portraits and judged them as more ambiguous (Boccia et al., 2014); also, content-dependent brain area of the ventral visual stream selectively activated during face perception (i.e., fusiform face area) was less activated when participants enjoyed Arcimboldo’s ambiguous portraits (Boccia et al., 2015). Overall, this evidence suggests that esthetic pleasure experienced during watching ambiguous Arcimboldo’s portraits is connected more with local processing of the objects than with global processing of the face.

On the one hand, this result suggests that an interaction between perceptual oscillation within the attributional network and attentional bias towards the local versus global level of percept occurs. On the other hand, this result suggests that artistic ambiguity is a useful tool in the cognitive neuroscience of art, especially with regard to the assessment of the attributional and attentional processes underlying esthetic experience of complex artworks.

Hierarchical letters offer also a good tool to test the effect of the attentional bias towards the local versus global level of percept. Gao et al. (2011) used the composite-face illusion and Navon stimuli to test the effect of local or global priming on subsequent face recognition; they found that allocating attention to the global level of prime (i.e., Navon stimuli) increased the tendency to process faces holistically. Here, we used a similar paradigm in order to investigate whether local and global prime affected esthetic appreciation of artworks showing whole-part ambiguity. To this aim we asked participants to detect target letters that might appear at a local (local prime) or global (global prime) level of hierarchical letters; immediately after, they judged Arcimboldo’s ambiguous artworks on a visual analogue scale (VAS). As a corollary, we also investigated whether such an effect interacted with participants’ perceptual style (PS). Thus, we also assessed participants’ PS using a classical Navon task. Based on the previous studies mentioned above, which suggest that biasing the orientation of visual attention towards the local level of percept would prompt the esthetic pleasure arising from watching ambiguous artworks, we predicted that local prime increased esthetic appreciation of ambiguous artwork and that this effect was further affected by PS.

## Method

### Participants

One hundred and forty-two individuals took part in this study. Three participants were excluded due to missing values in one of the two experimental conditions (further details are provided below in the experimental task description). Thus, the final sample included 139 healthy young individuals (age range: 20–35 years; mean age: 23.760 years; *SD*: 3.425; 91 women). Sample size was defined a priori using G*Power (Version 3.1.9.2) (Faul, Erdfelder, Lang, & Buchner, 2007) to achieve a statistical power higher than 80%, considering an alpha of 0.05. Mean and *SD* for determining the effect size (*d* = 0.225) was derived from a pilot study on six participants: average esthetic appreciation after *local prime* in pilot sample was 486.91 (*SD*: 252.16), whereas average esthetic appreciation after *global prime* was 475.03 (*SD* 258.82). None of the participants had a history of neurological or psychiatric disorders. All of them signed a consent form before the study began. This study was approved by the local ethics committee.

### Procedure

#### Questionnaire on art interests

At the beginning of each session, art interests were investigated using a modified version of the Questionnaire on art interests (Furnham & Chamorro-Premuzic, 2004).

#### Experimental task: Stimuli and procedure

Stimulus presentation and response collection were controlled by scripts (OpenSesame 3.2.7; Mathôt, Schreij, & Theeuwes, 2012) running on a PC desktop computer with a Windows 10 operating system. The stimuli were presented on a 15.6-in. computer screen (1,366 × 768 pixel resolution).

#### Experimental task: Local versus global prime on esthetic appreciation of ambiguous artworks

Twelve of Arcimboldo’s artworks (Table 1) were presented twice in a random order, preceded by hierarchical letters. The artworks measured 500 × 750 pixels (width × height) and were projected in the center of the screen. The hierarchical letters consisted of global Es (i.e., global prime) composed of local Hs, Fs, or Ts, or global Hs, Fs, or Ts composed of local Es (i.e., local prime); they measured 250 × 400 pixels (width × height) and were projected in the center of the screen.

**Table 1:** Arcimboldo’s ambiguous portraits. Stimuli were derived from the larger set used in previous studies (Boccia et al., 2014, 2015, 2017) as those receiving similar esthetic evaluation of non-ambiguous Renaissance portraits (original data were published in Boccia et al., 2014)

Painting	Year
Autumn	1563
Autumn	1573
Flora	1589
Librarian	1566
Portrait of Adam	1578
Portrait of Eve	1578
Spring	1563
Spring	1573
Summer	1563
Summer	1573
Vertumno	1591
Winter	1563

Each trial started with a green fixation point (500 ms) followed by a red fixation point (500 ms), which signaled the incoming of a hierarchical letter (Fig. 1). Participants were asked to lay their index and middle fingers on the M and K keys of the keyboard and instructed to press M if the letter E appeared at the local level and K if the letter E appeared at the global level. Hierarchical letters remained on the screen until participants provided the answer or for a maximum time of 2,500 ms. Immediately afterward an Arcimboldo’s artwork was presented along with a computerized VAS (line range = 0–1,024 pixels). Participants were asked to point the cursor along the VAS and click the left button to indicate their esthetic appreciation; they were asked to answer as soon as possible; position and response time were automatically registered. The main experiment was preceded by a practice phase in which participants performed the same tasks described above on a different set of artworks.

Trials in which response time was lower than 500 ms or higher than 5,000 ms on the VAS were excluded. Also, we excluded trials when the level at which the target letter appeared (i.e., local vs. global) was not correctly identified; unfortunately, three participants failed to provide correct responses in one of the two conditions, and thus they were excluded from the analyses. Thus, for each participant and condition (i.e., local and global prime), the mean VAS score was calculated.

#### Perceptual style (PS) classification: The Navon task

Here we used a modified version of the Navon Task we developed in a previous study (Boccia et al., 2014). Target stimuli included global letters that were either Es composed of local Fs, Hs, or Ts, or global Hs, Fs, or Ts composed of local Es. Nontarget stimuli were global Fs, Hs, Ls, or Ts composed of local Ls, Hs, Ts, or Fs. The global letters measured 250 × 400 pixels (width × height) and were presented in the center of the computer screen, three times per participant, in a randomized order.

**Fig. 2:**
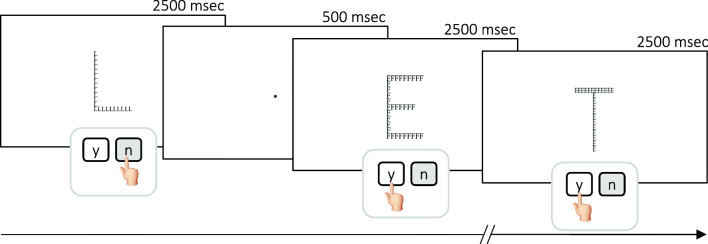
*Navon task*: Timeline and examples of stimuli. *Notes* y = yes; n = no

Participants were asked to lay their index and middle fingers on the M and K keys of the keyboard and instructed to press M every time the letter E appeared, at either the global or the local level (target condition) and to press K if other stimuli appeared on the screen (Fig. 2). The stimuli remained on the screen until one of the two response keys had been pressed or for a maximum time of 2,500 ms. A practice phase preceded the experimental task. Both accuracy and response times were registered.

For each participant and condition, we calculated the perceptual preference index on accuracy (aPI) using the formula:

$$ aPI=\frac{nL}{\left( nL+ nG\right)} $$

where *n* is the number of correct answers on local (*L*) or global (*G*) level of the Navon task. *aPI* higher than 0.5 corresponded to local preference (i.e., higher rate of correct responses on local level of the Navon task), whereas *aPI* lower than 0.5 corresponded to global preference.

To disambiguate between local and global preference in participants whose *aPI* was equal to 0.5, we computed an additional perceptual preference index: for each participant, we computed the average response times of correctly performed trials of the Navon task; thus, similar to our previous study (Boccia et al., 2014), we computed the difference between mean response times of local and global targets in the Navon task as it follows:

$$ I= rtL- rtG $$

where *I* is the difference between response times (rt) on local (*L*) and global (*G*) level of the Navon task. If *I* was higher than 0, then the participant was faster at the global level, and if *I* was lower than 0, then the participant was faster at the local level. Thus, *I* higher than 0 corresponded to global perceptual preference, whereas *I* lower than 0 corresponded to local perceptual preference.

Thus, participants were classified as having local PS if *aPI* was higher than 0.5 or if their aPI was equal to 0.5 but they were faster on local level of the Navon task (I < 0). Otherwise, participants were classified as having global PS if their aPI was lower than 0.5 or if their aPI was equal to 0.5 but they were faster on global level of the Navon task (I > 0).

### Statistical analyses

Statistical analyses were run using SPSS 25. As a first step of our analysis, we tested whether groups of participants with local and global PS were matched for age, gender, and art interests, by means of two-sample t-tests.

Thus, we tested whether global and local prime affected esthetic appreciation (VAS score) of Arcimboldo’s artworks by means of a paired t-test. Also, we tested whether global and local prime differently affected esthetic appreciation of participants with local and global PS, by means of a mixed-factorial ANOVA, with Group (local PS vs. global PS) as between-group factor and Prime (local vs. global) as within-group factor. The same set of analyses was performed on response times of esthetic appreciation.

## Results

Preliminary analyses on demographics suggested that there was no clear indication of a difference between participants with global and local PS for age (*t*(137) = 0.008; *p* = 0. 993), gender (*χ*^*2*^ = 2.544; *p* = 0.111), and artistic interests (*t*(137) = 0.243; *p* = 0. 809).

A paired t-test on esthetic appreciation of Arcimboldo’s artworks preceded by local or global prime revealed a significant difference (*t*(138) = -2.477; *p* =0. 014; Cohen’s *d* = 0.210), with higher appreciation when the artworks were preceded by local (mean: 664.619; *SD*: 224.864) than global (mean: 644.727; *SD*: 228.589) prime.

**Fig. 3:**
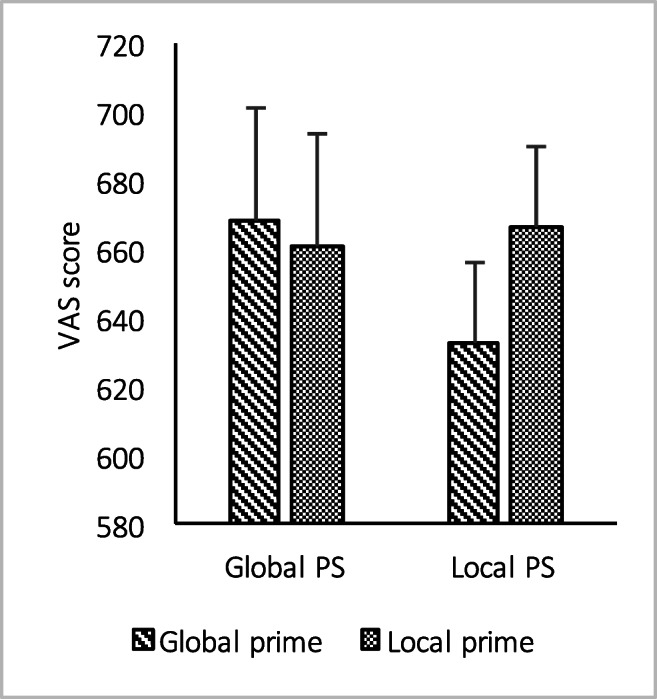
Bar graph depicts Group (local vs. global PS) by Prime (local vs. global) interaction on esthetic judgment assessed by means of VAS. Bars depict mean and standard error. *Notes* PS = perceptual style; VAS = visual analogue scale

Mixed-factorial ANOVA (Levene’s test for homoscedasticity: p > 0.302; Sphericity: Mauchly’s W = 1.00) revealed a Group by Prime interaction on esthetic appreciation (*F*(1,137) = 6.232; MS = 26922.139; *p* = 0.014; *η*_*p*_^*2*^ = 0.044; observed power = 0.698): post hoc pairwise comparisons showed that only participants with local PS differed on local versus global prime (*p* = 0.001, Bonferroni’s correction for multiple comparisons was applied), with a lower rating for globally primed artworks (Fig. 3). Main effects of Group (*F*(1,137) = 0.139; MS = 13,750.093; *p*= 0.710; *η*_*p*_^*2*^ = 0.001; observed power = 0.066) and Prime (*F*(1,137) = 2.493; MS = 10,771.202; *p* = 0.117; *η*_*p*_^*2*^ = 0.018; observed power = 0.348) were not significant.

With regard to response times, a paired t-test revealed only a trend towards significance (*t*(138) = -1.932; *p* = 0.055; Cohen’s *d* = 0.125) with response times slightly slower for local prime (M = 2,089.463; *SD* = 494.762) than global prime (M = 2,028.059; *SD* = 490.700). Mixed factorial ANOVA on response times (Levene’s test for homoscedasticity: p > 0.322; Sphericity: Mauchly’s W = 1.00) failed to reveal any significant effect (Main effect of Prime: *F*(1,137) = 2.726; MS = 192,364.615; *p* = 0.101; *η*_*p*_^*2*^ = 0.020; observed power = 0.374; Main Effect of Group: *F*(1,137) = 0.025; MS = 10,455.520; *p* = 0.875; *η*_*p*_^*2*^ = 0.000; observed power = 0.053; Group by Prime interaction: *F*(1,137) = 0.283; MS = 19,958.116; *p* = 0.596; *η*_*p*_^*2*^ = 0.002; observed power = 0.083).

## Discussion

Here we investigated whether local and global prime affected esthetic appreciation of ambiguous artworks showing whole-part ambiguity and whether this effect interacted with perceptual style. Our results pointed towards two key findings: first, local prime yielded to higher rate of esthetic appreciation of Aricimboldo’s ambiguous portraits. Second, effect of prime interacted with perceptual style: indeed, only participants with local perceptual style differed on local versus global prime, showing a lower rate of esthetic appreciation for globally primed artworks as compared with locally primed ones.

The present results are consistent with previous findings about local processing of Arcimboldo’s ambiguous portraits. Previous studies have suggested indirectly that ambiguous portraits showing whole-part ambiguity were judged more beautiful when processing of local parts prevailed on global processing of the face (i.e., the whole). This indirect evidence comes from neuroimaging results (Boccia et al., 2015) and the effect of individual differences due to the perceptual style (Boccia et al., 2014). The present study directly tested the effect of local versus global redirection of attention by using hierarchical stimuli such as Navon’s letters as a prime, similar to previous studies (Gao et al., 2011; Poirel et al., 2014), providing additional evidence about the positive effect of local processing on esthetic appreciation of Arcimboldo’s ambiguous portraits. Looking at a painting, perception is usually dominated by the whole spatial layout. Thus, it is difficult to see the details within, although effort and purposeful scanning allow for overcoming the attraction to the conglomerate presentation to see the local features (Zaidel, 2015). We may speculate that local prime facilitates focusing on local parts and allows for seeing the details within the ambiguous portraits, thus increasing appreciation and strengthening our fascination for the holistic processing of the whole, namely the face. Based on evidence that perceptual challenge increases esthetic appreciation (Muth & Carbon, 2013), we may also hypothesize that priming local scanning may result in greater insight and elaboration, which prompt the Esthetic Aha effect.

Response times for local prime seem to differ from those observed for global prime only slightly. Further interpretations about the effect of local and global prime on timing of esthetic appreciation deserve future investigations. It would be interesting to test whether such an effect occurs during the early phase of esthetic experience, namely during esthetic appreciation sensu stricto, or later, during esthetic appreciation sensu lato. Here, we cannot draw conclusions about the stage at which this interaction takes place: we asked participants to answer as soon as possible and also dismissed responses occurring after 5,000 ms to avoid further reflection on interest and originality of the artworks; thus, we can hypothesize that this effect occurs likely during esthetic appreciation sensu stricto. However, according to the results by Cela-Conde et al. (2013), esthetic appreciation sensu stricto occurs between 250 ms and 750 ms, whereas esthetic appreciation sensu lato occurs between 1,000 ms and 1,500 ms. The use of VAS to assess individuals’ esthetic judgment makes it impossible to restrict the response-time window to these intervals. This is also evident from average data on response times for local and global prime. Thus, further studies, by using two alternative forced choice tasks (e.g., like vs. dislike), should test the interesting idea that the effect of hierarchical prime and of the perceptual style may occur in specific time spans of the esthetic evaluation process.

More in general, the present results provide useful insights into the theoretical models of esthetic experience and perception. We found that local versus global prime affected participants with local and global perceptual style, differently: indeed, we observed a decrease of esthetic rating for portraits preceded by global prime in participants with local perceptual style. No effect was detected for participants with global perceptual style. On the one hand, this result suggests that global prime, likely contrasting perceptual facilitation for local level of hierarchical stimuli in participants with local perceptual style, yielded to a dramatic decrease of esthetic judgment. On the other hand, this result ties well with the results of inhibitory control during local processing by Poirel et al. (2014): using a negative priming paradigm, these authors found that global-to-be-ignored information negatively affected execution of the local condition of the Navon task; instead, no effect was observed for local-to-be-ignored information during execution of a global condition of task. In this vein, a significant decrease of appreciation for a global non-preferred prime may be due to negative interference of this condition on local-preferred processing. Instead, no effect was detected for local non-preferred prime, since this condition did not affect global processing (Poirel et al., 2014).

Some caution needs to be exercised with regard to the present study, especially with regard to the possible generalization of the results. Part-whole ambiguity of Arcimboldo’s artworks offers a good tool for testing our experimental hypothesis without spurious perceptual effects. However, it restricts any possible generalization of our results to other types of perceptual complexity and ambiguity in art. Also, the uniqueness of the stimuli used in our study, namely Arcimboldo’s ambiguous portraits, prevents any possible generalization of our results to other categories of paintings. Thus, future studies using different set of artworks are mandatory to draw definite conclusions. Also, more fine-grained manipulation of non-artistic stimuli (see, e.g., Austen & Enns, 2000) may help in disentangling the interaction between attentional and perceptual processing during esthetic appreciation. Finally, further studies should test the effect of local and global prime on the highest levels of esthetic appreciation, such as those involving emotional systems.

Overall, the present findings shed some light on the processes involved in esthetic experience, pointing towards a pivotal role of re-direction of attention towards perceptual features of artworks, and open new interesting questions about this complex process.

### Open practices statement

The data and materials for all experiments are available from the corresponding author upon request. The present study was not preregistered.
